# Bad Water, Filth and Typhoid Fever

**Published:** 1889-02

**Authors:** 


### BAD WATER, FILTH, AND TYPHOID FEVER.

Lakeview, a suburb of Chicago, has had a very large number of cases of typhoid fever during the past few weeks, and at present it is said that there is an epidemic of the disease in the town. Unfortunately for those that wish to get at the facts, local politics seems to figure very prominently in the matter, one side suppressing or ignoring the facts, while the other side appears to exaggerate them. A sifting of all the evidence, however, shows that the water supply of Lakeview is bad; that the town is in a deplorable sanitary condition, that a very large number of cases of typhoid fever exist in the town, and that the mortality has been large. In 1888 the number of deaths from typhoid fever in Lakeview was 18, the number in January, 1889, was 23, in a population estimated at 50,000. To put the matter in dollars, typhoid fever cost Lakeview in 1888, in deaths alone $14,310, while in the last month alone it cost the town $18,285. This is exclusive of the cost of cases of illness.

A careful review of such facts as can be had seems to show that bad water and the filthy condition of the town are the causes of the outbreak of typhoid fever in Lakeview, and makes it probable that the health authorities of the town are not blameless. The whole number of cases of the disease reported in January was 97; with 23 fatal cases, the death-rate was a little more than 23 per centum, showing that the disease was of unusual virulence.

In regard to the water-supply of Lakeview, during the latter part of December the inlet-pipes from Lake Michigan became clogged with ice. These pipes are laid 2,000 and 2,200 feet out into the lake. The water engineer, instead of having this ice removed, began to pump in water from the shore inlet, within the breakwater and only a short distance from the shore—about 150 feet. This was done for about a month, and during that time the inhabitants of Lakeview have been using water contaminated by sewage. Added to this, if anything need be added, the alleys and streets of Lakeview are, and they have been for several months, in a filthy condition. There is the possibility, also, that, in the absence of adequate inspection of milk, at least some of the milk used by the people of Lakeview has been diluted by this same impure water. The inadequacy of the Lakeview water-pipes to the purpose for which they were laid is shown by the fact that this, one of the warmest winters on record at this place, has sufficed to clog the pipes with ice. Perhaps with less of politics and jobbery in municipal offices the health of some of our cities might be improved.

